# Diversifying the platinum-based chemotherapy toolkit for immunogenic cancer cell death

**DOI:** 10.18632/oncotarget.27713

**Published:** 2020-09-08

**Authors:** Abhishek D. Garg, Patrizia Agostinis

**Keywords:** PT-112, immunogenic cell death (ICD), immune-checkpoint blockers (ICBs), tumour microenvironment, apoptosis

Platinum-based chemotherapeutics, such as cisplatin, oxaliplatin and carboplatin, are amongst the most widely applied anticancer agents, with well-established efficacy against several cancer-types [1, 2]. In fact, in few clinical settings (e.g., loco-regionally advanced cancer and metastatic germ cell cancers), platinum-based chemotherapy has exhibited promising curative effects, when combined with other conventional anticancer modalities [1]. The binding of reactive platinum molecules derived from these chemotherapies (after they enter the cells) to DNA, thereby creating potent platinum–DNA adducts, is widely considered to be the primary *modus operandi* behind their anticancer activity [3]. Such adducts interfere with normal DNA turn-over and repair machinery, thereby inducing regulated cell death (RCD) mainly in the form of apoptosis [3].

For a long-time it was considered that the mechanistic underpinning of the anticancer efficacy of such platinum-based chemotherapies originated from their ability to induced DNA damage causing cancer cell death [3]. However, over the last decade, it has been repeatedly documented that these platinum-based chemotherapies can also enhance the immunogenicity of dying cancer cells by inducing immunogenic cell death (ICD), in a context-dependent fashion [4]. ICD is a form of (apoptotic or necroptotic) RCD that exhibits immunogenic characteristics due to the exposure and delivery of various immunomodulatory signals e.g., damage-associated molecular patterns (DAMPs), which stimulate the innate immune cells to prime adaptive immune responses against tumor antigens [4, 5]. Cancer cells undergoing ICD can elicit potent anticancer immunity, predominantly in cancer cells enriched in carcinogen-induced antigens [5]. Interestingly, qualitative as well as quantitative differences have been observed between different platinum-based chemotherapies in terms of ICD-induction capacities [4]. For instance, while oxaliplatin has the most consistent ICD-inducing capacity yet cisplatin/carboplatin tend to induce largely non-ICD characteristics [4]. Although in some exceptional instances, cisplatin can also exhibit ICD-like activity (the mechanistic basis of which, is as-yet unclear) [6]. Nevertheless, a clear chemical structure-function relationship between these agents and ICD-induction has not been established thereby mandating systematic testing of all platinum-based chemotherapies in order to expand the ICD toolkit. Interestingly, several new platinum-based chemotherapeutic agents are currently entering clinical oncological trials. One such promising agent is R,R-1,2 cyclohexanediamine-pyrophosphato-platinum(II) (PT-112) [7]. PT-112, owing to a more robust chemical structure, has better pharmacokinetic/pharmacodynamic properties compared to currently used platinum-based chemotherapeutics, which accounts for its improved anticancer effects [7]. In line with this, preliminary measurements of clinical benefit in cancer patients resistant to various conventional and/or experimental modalities, showed some early (therapeutic) benefits upon PT-112 treatment within ongoing Phase I clinical trials (NCT02266745, NCT03409458) – although these observations need to be further verified in advanced clinical trials [7]. Nevertheless, PT-112 monotherapy was also reported to exhibit anticancer efficacy in two patients with resistance toward immunotherapy [7]. Although these observations underscore the promising anticancer efficacy of PT-112 yet they also hint toward its possible pro-immunogenic activity. However, little is known about the cell death-associated immunogenic characteristics of PT-112 in the setting of anticancer therapy.

Interestingly, a recent study by Yamazaki et al. tried to address this gap-in-knowledge [7]. Yamazaki et al. documented that PT-112 exhibits promising *in vitro* cell death-inducing activity against several human and murine cancer cell lines [7]. In line with a ICD-like profile, the cell death induced by PT-112 associated with the main ICD-associated DAMPs like calreticulin surface exposure, ATP secretion and passive HMGB1 release [7]. Moreover, Yamazaki et al. were able to successfully demonstrate that cancer cells dying in response to PT-112 *in vitro*, induced anticancer vaccination effect *in vivo*; i.e., when PT-112 treated dying/dead cancer cells were injected as “vaccines” in syngeneic, immunocompetent mice, these mice developed immunological immunity against a consequent challenge with live cancer cells of identical-type [7]. These results establish PT-112 as a new platinum-based chemotherapeutic with ICD-inducing capacity.

Over the years, immunotherapy involving immune-checkpoint blockers (ICBs) that inhibit immune-checkpoint signalling downstream of PD1/PD-L1 or CTLA4, has become standard-of-care for several cancer-types [8]. Hence currently, it is a ‘hot area’ of investigation to characterize chemotherapies that can (or cannot) synergize with ICBs. Importantly, at least one clinical trial is currently exploring combination of PT-112 with the PD-L1 targeting ICB, Avelumab (NCT03409458), thereby making it imperative to preclinically test the immunological effects of the combination of PT-112 with ICB. To address this, Yamazaki et al. administered tumour-bearing mice with PT-112 in combination with different ICBs and found that PT-112 synergizes with ICBs targeting PD1 or PD-L1 in reducing tumour burden (in immune-competent settings) [7]. These anticancer effects in combinatorial settings were accompanied by intra-tumoural recruitment of various effector immune cells paralleling a reduction in immunosuppressive cells [7]. Interestingly, the combination of these ICBs with PT-112 also exhibited “abscopal effect” i.e., treatment of a particular tumour reduced the growth of another distant (untreated) tumour [7].

Altogether these results demonstrate that, PT-112 not only exerts pro-immunogenic effects via ICD but can also act as a promising combinatorial agent for ICB-based immunotherapy. Of note, the *in vivo* immunological assays in this study were mainly performed with relatively immunogenic cancer models like TS/A murine breast cancer cells or CT26/MC38 murine colon cancer cells, that have been widely used to study and annotate ICD [7]. In future it would be interesting to explore how PT-112 performs against highly immunoevasive or negligibly immunogenic cancer models where standard ICD frequently fails. Additionally, further investigations on the molecular underpinnings of the danger signaling pathways elicited by PT-112 would be instrumental in understanding why assorted platinum-based chemotherapies induce ICD, while others like cisplatin, are much less effective. Nevertheless, the observations of Yamazaki et al. are intriguing and create a definitive incentive to further explore the exact cellular and molecular mechanisms underlying their results. Such insights can help in conceptualization of more improved platinum-based chemotherapies with augmented pro-immunogenic effects against tumours.
